# First-Principles Dynamics Investigation of Germanium as an Anode Material in Multivalent-Ion Batteries

**DOI:** 10.3390/nano13212868

**Published:** 2023-10-30

**Authors:** Chaewon Kim, Useul Hwang, Sangjin Lee, Young-Kyu Han

**Affiliations:** Department of Energy and Materials Engineering, Dongguk University-Seoul, Seoul 04620, Republic of Korea; twinstees@dongguk.edu (C.K.); bbohyunh@gmail.com (U.H.)

**Keywords:** germanium, multivalent-ion battery, magnesium, volume expansion, anode material

## Abstract

Germanium, a promising electrode material for high-capacity lithium ion batteries (LIBs) anodes, attracted much attention because of its large capacity and remarkably fast charge/discharge kinetics. Multivalent-ion batteries are of interest as potential alternatives to LIBs because they have a higher energy density and are less prone to safety hazards. In this study, we probed the potential of amorphous Ge anodes for use in multivalent-ion batteries. Although alloying Al and Zn in Ge anodes is thermodynamically unstable, Mg and Ca alloys with Ge form stable compounds, Mg_2.3_Ge and Ca_2.4_Ge that exhibit higher capacities than those obtained by alloying Li, Na, or K with Ge, corresponding to 1697 and 1771 mA·h·g^–1^, respectively. Despite having a slightly lower capacity than Ca–Ge, Mg–Ge shows an approximately 150% smaller volume expansion ratio (231% vs. 389%) and three orders of magnitude higher ion diffusivity (3.0 × 10^−8^ vs. 1.1 × 10^−11^ cm^2^ s^−1^) than Ca–Ge. Furthermore, ion diffusion in Mg–Ge occurs at a rate comparable to that of monovalent ions, such as Li^+^, Na^+^, and K^+^. The outstanding performance of the Mg–Ge system may originate from the coordination number of the Ge host atoms and the smaller atomic size of Mg. Therefore, Ge anodes could be applied in multivalent-ion batteries using Mg^2+^ as the carrier ion because its properties can compete with or surpass monovalent ions. Here, we report that the maximum capacity, volume expansion ratio, and ion diffusivities of the alloying electrode materials can be understood using atomic-scale structural properties, such as the host–host and host–ion coordination numbers, as valuable indicators.

## 1. Introduction

Advancements in electrochemical energy storage technology led to extensive research on various systems, such as lithium-ion batteries, Li–S batteries, Li–Se batteries, aqueous ammonium-ion batteries, aqueous Zn-ion batteries, and supercapacitors [1,2,3,4,5,6,7]. Among these, multivalent-ion batteries (MIBs) are receiving considerable attention as promising alternatives to lithium-ion batteries (LIBs), owing to their earth abundance and cost efficiency. The ability of divalent (Mg^2+^, Ca^2+^, and Zn^2+^) and trivalent (Al^3+^) ions to transfer more than one electron allows them to store more energy in batteries than is possible with monovalent LIBs [8,9,10]. Moreover, double- or triple-electron exchange per ion during the electrochemical reaction could potentially yield higher specific energy densities [8,11]. Metal negative electrodes such as Al, Mg, Ca, and Zn are considered extremely favorable since they can provide remarkably high gravimetric (820–2980 mA·h·g^–1^) and volumetric capacities (2073–8046 mA·h·cm^–3^), which are substantially higher than the 372 mA·h·g^–1^ and 818 mA·h·cm^–3^, respectively, of the graphite anode used in commercial LIBs [11,12,13,14,15,16]. However, using metal anodes presents several unfavorable issues, including sluggish ion diffusion kinetics in both the electrolyte and electrodes, instability of the electrodes, formation of a complicated solid electrolyte interface, and self-corrosion of the anodes [17,18,19]. Certain anode materials, such as titanium oxides and vanadium oxides were investigated based on their intercalation reactions [20,21]; however, research on anodes for MIBs remains challenging compared to that on cathodes. Despite the promise of emerging materials, such as organic electrodes [22] and structure-engineered composites [23], which have advantages that include flexibility in design and a wide range of property tunabilities, the practical implementation of MIBs was hindered by inherent challenges, including relatively low redox potentials, and insufficient electronic and ionic conductivities.

One material that was widely investigated as a highly promising material for the next generation of anodes in LIBs is germanium (Ge) [21,24,25,26,27] because of its high specific capacity (1624 and 1384 mA·h·g^−1^ for Li_22_Ge_5_ and Li_15_Ge_4_ alloys, respectively [28]), superb rate performance, and good cycling reliability due to its fast kinetics for both Li^+^ ion diffusion and electronic conduction [27,29,30,31]. However, as with other alloy-based materials used in negative electrodes in LIBs, Ge undergoes large volume changes in the fully lithiated state of Li_22_Ge_5_. Numerous strategies were suggested to overcome this problem [25,32], and these strategies will likely enhance the electrochemical performance of Ge anode materials in LIBs in the near future.

Both Ge and Si anodes received significant attention as next-generation LIB anodes. They share several similarities, including the obstacle of having a large volume expansion ratio upon lithiation, which can result in capacity fading. However, Ge anodes have several advantages over Si anodes. First, the capacity of Ge is only half that of Si (1624 vs. 3579 mA·h·g^−1^); however, it has a higher density (5.30 vs. 2.30 g·cm^−1^), which endows it with a charge stored per volume similar to that of Si [25,33]. Second, the Li-ion diffusivity is two orders of magnitude quicker for Ge (at room temperature, 6.51 × 10^–12^ vs. 1.41 × 10^–14^ cm^2^·s^–1^) than for Si. Third, the electronic conductivity is three orders of magnitude higher for Ge than for Si (2.1 vs. 1.6 × 10^–3^ S·m^–1^) [34,35]. Thus, an analysis of the advantages of Ge anodes is essential for the development of next-generation LIB anodes.

Exploring amorphous Ge anodes for MIBs is also a worthwhile research endeavor. While a low reversible capacity less than 350 mA·h·g^–1^ was reported for the crystalline Ge anode in Na-ion batteries [36], our group theoretically suggested [37,38] that Ge becomes reactive toward Na ions when its amorphous phase is adopted, thereby providing a maximum capacity of 576 mA·h·g^–1^ at Na_1.56_Ge. After the publication of our computational results, several experimental studies [39,40] performed on amorphous Ge anodes reported initial reversible capacities of ≤430 mA·h·g^–1^.

Considering the studies on Na-ion batteries, amorphous Ge may be a promising candidate for utilization as the anode in MIBs, as it has the potential to offer higher capacities than crystalline Ge. However, the diffusion kinetics are poorer for multivalent ions than for Li ions owing to the stronger coulombic interactions between multivalent carrier ions and the electrode material [41,42]. This sluggish and poor diffusion of multivalent ions was primarily observed in crystalline electrodes, particularly in metal oxides, such as Ni_2_O_4_, Mn_2_O_4_, TiO_2_, and K*_x_*W_3_O_9_ [10]. We believe that higher ion diffusivity will be achieved with amorphous Ge than with crystalline Ge. Unlike crystalline Ge, amorphous Ge does not have long-range order [43,44]; therefore, it can accelerate the structural opening of the localized transport pathway through which multivalent ions migrate.

First-principles molecular dynamics (FPMD) simulations can provide atomistic structural descriptions of amorphous materials with precision at the quantum mechanical level. This enables the derivation of specific values for several parameters, such as atomic coordination numbers, bond angles, and interatomic distances in the structures of amorphous materials, which are difficult to determine experimentally. This distinctive information can be applied to examine the diffusion properties and reaction processes of carrier ions in amorphous electrodes using FPMD simulations. FPMD simulations were used by our group to provide a clear description of amorphous negative electrodes, such as crystalline Si/amorphous Li*_x_*Si interfaces [45], amorphous Si anodes for Na [37], K [46], and Mg [47], and amorphous Al_2_O_3_ interfaces for Li and Na [48,49]. In addition, we were able to reproduce and provide a reasonable explanation for the findings of several experimental studies on these systems [50,51,52]. Assessing the electrochemical performance by investigating the reactions of amorphous Ge materials with various multivalent ions via FPMD simulations may provide experimental researchers with research strategies for fabricating MIB anodes.

We performed FPMD simulations to gain insights into the chemical bonding and structural changes that occur when amorphous Ge material alloys with the multivalent Mg^2+^ and Ca^2+^ cations. We found that the Mg–Ge and Ca–Ge alloys were most stable at compositions of Mg_2.3_Ge and Ca_2.4_Ge, respectively, and showed the highest capacities of 1697 and 1771 mA·h·g^–1^, respectively. Although the Mg_2.3_Ge alloy has a slightly lower specific capacity than Ca_2.4_Ge (~4%), it exhibits a ~150% smaller volume expansion ratio and a three orders of magnitude-higher ion diffusivity than Ca_2.4_Ge, while demonstrating comparable or superior values to those of Li_4.0_Ge, Na_1.5_Ge, and K_1.2_Ge. We found that the values of the average coordination numbers of CN_host–host_ and CN_host–ion_ are decisive descriptors for comprehending the specific capacity, volume expansion, and ion diffusivity of alloy-based negative electrode materials. The findings of this study suggest that the Mg^2+^ cation has the highest potential among the multivalent ions for use as a promising carrier ion in Ge anodes, and the performance of Mg–Ge alloys is comparable to that of monovalent ions.

## 2. Computational Details

First-principles density functional theory (DFT) computations were carried out as implemented in the Vienna ab initio simulation package (VASP). The Perdew–Burke–Ernzerhof (PBE) exchange and correlation functionals and the projector augmented wave (PAW) method were adopted. The valence electron configurations considered for Li, Na, K, Al, Mg, Ca, Zn, and Ge were 1s^2^2s^1^, 2p^6^3s^1^, 3p^6^4s^1^, 3s^2^3p^1^, 2p^6^3s^2^, 3p^6^4s^2^, 3d^10^4s^2^, and 4s^2^4p^2^, respectively. In our study, we constructed a periodic cubic super cell with 40 Ge atoms 40 × *x* M atoms for each amorphous M*_x_*Ge (M = Li, Na, K, Mg, Al, Ca, and Zn) structure. A 3 × 3 × 3 k-point mesh was used for the Brillouin zone integration. The simulation procedure for amorphous M*_x_*Ge (M = Li, Na, K, Mg, Ca, Zn, and Al) alloys consists of two steps: (1) volume relaxation and (2) energy evaluation. First, for a given ion concentration (*x*), the volume of M*_x_*Ge was determined using the liquid quench method. Initially, the cubic supercells containing 40 × *x* M and 40 Ge atoms were randomly generated with estimated dimensions. This structure underwent a heating process (up to 2000 K, 1 K·fs^–1^), followed by equilibration (at 2000 K for 3 ps), and was finally quenched (down to 300 K, 1 K·fs^–1^) [53,54]. The quenched structure was equilibrated for an additional 5 ps at 300 K (Figure S1). From the last 2 ps of the equilibrating run, five structures were picked for every 500 computation steps (Figure S1) and then fully relaxed until the atomic position, supercell shape, and supercell volume were optimized as the residual force converged within 0.03 eV·Å^–1^. In this step, the plane-wave kinetic energy was augmented by 30% to mitigate the Pulay stress problem [55]. The detailed FPMD simulation procedure is included in the Supporting Information.

For amorphous Ge, the computed physical and chemical properties are in close accordance with those reported in previous studies. The formation energy is 0.11 eV higher per Ge atom for amorphous Ge than for crystalline Ge. The predicted density of amorphous Ge (5.57 g·cm^–3^) is comparable to the experimental result (5.32 g·cm^–3^ [33]). The simulated Ge–Ge bond length (2.46 Å), analyzed by the radial distribution functions (RDFs), is consistent with 2.3–2.5 Å in theoretical [56] and experimental studies [57,58]. The agreement between the calculated and experimental results supports the accuracy of the amorphous structures obtained from our FPMD simulations.

## 3. Results and Discussion

**Figure 1:**
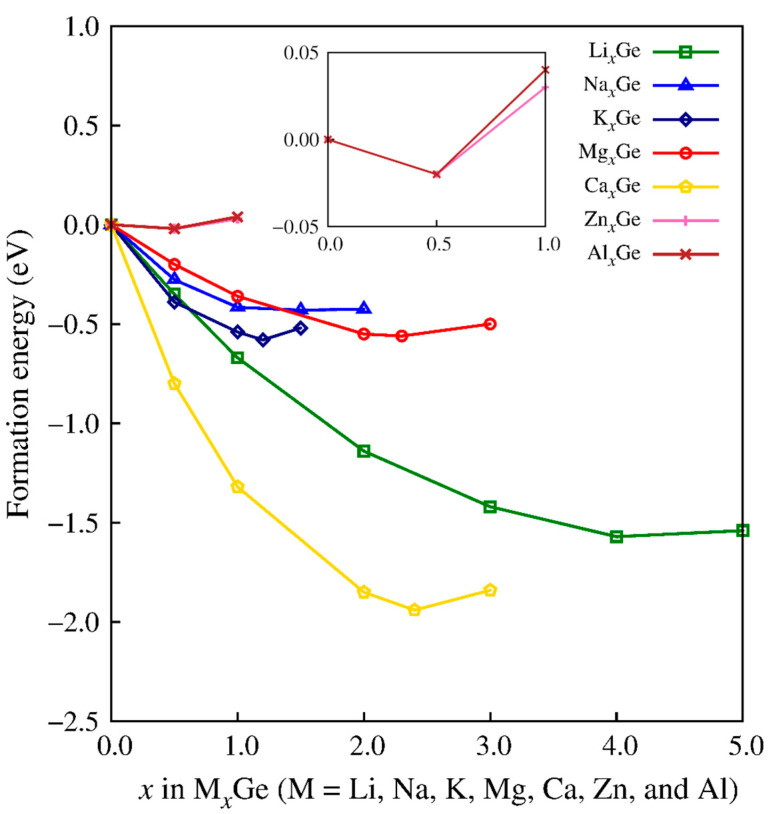
Formation energies of the M*_x_*Ge (M = Li, Na, K, Al, Mg, Ca, and Zn) amorphous alloys.

Mg–Ge, Ca–Ge, Al–Ge, and Zn–Ge amorphous alloys were generated using FPMD simulations. Figure 1 shows the formation energies of amorphous Mg*_x_*Ge, Ca*_x_*Ge, Al*_x_*Ge, and Zn*_x_*Ge (0.0 ≤ *x* ≤ 5.0) calculated with a reference to an amorphous Ge electrode. The formation energies were positive for the Zn–Ge, and Al–Ge alloys, whereas those for the Mg–Ge, Ca–Ge, Li–Ge, Na–Ge, and K–Ge alloys had negative values. These results indicate that the electrochemical reactions required for Mg^2+^, Ca^2+^, Li^+^, Na^+^, and K^+^ ions to form alloys in amorphous Ge are thermodynamically favorable, whereas Zn^2+^ and Al^3+^ cannot alloy stably with amorphous Ge. The alloys with negative formation energies, such as the Mg–Ge, Ca–Ge, Li–Ge, Na–Ge, and K–Ge alloys, were also thermodynamically favorable against separation into Mg, Ca, Li, Na, and K metals and the Ge anode. The amorphous alloys of Mg–Ge and Ca–Ge that had the most stable ion concentration were Mg_2.3_Ge and Ca_2.4_Ge, corresponding to formation energies of −0.56 and −1.94 eV, respectively, and had high capacities of 1697 and 1771 mA·h·g^–1^, respectively.

We also compared the formation energies of Ge alloyed with divalent Mg^2+^ and Ca^2+^ to those of Ge alloyed with monovalent ions, as shown in Figure 1. The most stable ion concentrations for Li_4.0_Ge, Na_1.5_Ge, and K_1.2_Ge corresponded to formation energies of −1.57, −0.40, and −0.58 eV, respectively. The concentration of Li (*x* = 4.0) at the most stable state agreed well with previously reported Ge electrodes for lithium [24,34]. While a sufficiently low formation energy for metal electrode alloys is crucial to guarantee a high redox potential, high ion concentrations are also critical for batteries to provide sufficient capacities. The calculated specific capacities of the Li–Ge, Na–Ge, and K–Ge alloys were 1476, 553, and 443 mA·h·g^–1^, respectively, showing that the Li–Ge alloy had the highest capacity. Despite the relatively low stability and capacities of the Na–Ge and K–Ge alloys compared to those of the Li–Ge alloy, the negative formation energies would still enable Na^+^ and K^+^ ions to favorably alloy with Ge. The formation energies of the energetically most stable ion concentrations for the M*_x_*Ge (M = Mg, Ca, Li, Na, and K) systems suggested that the strength of the driving force for M–Ge alloying has an order of Ca > Li > K ~ Mg > Na. However, a noteworthy point is that the capacities were higher for Mg_2.3_Ge (1697) and Ca_2.4_Ge (1771 mA·h·g^–1^) than for Li_4.0_Ge (1476 mA·h·g^–1^).

**Figure 2:**
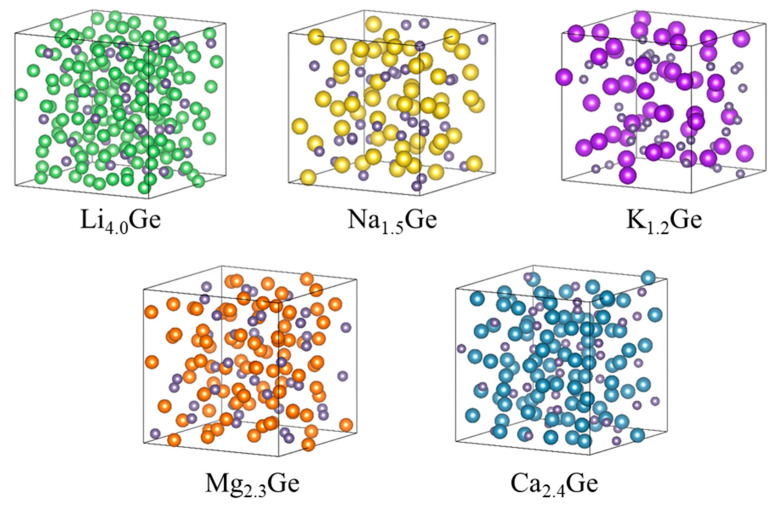
Atomic structures of M*_x_*Ge (M = Mg, Ca, Li, Na, and K), where *x* is at the maximum concentration. Green, yellow, purple, orange, blue, and gray spheres represent Mg, Ca, Li, Na, K, and Ge atoms, respectively.

Figure 2 shows the atomic structures of amorphous M*_x_*Ge (M = Mg, Ca, Li, Na, and K) (*x* = full composition). The compositions of Mg_2.3_Ge and Ca_2.4_Ge were generally consistent with the presence of Mg_2_Ge and Ca_2_Ge crystals in the Mg–Ge and Ca–Ge phase diagrams, respectively [59,60]. The formation energies of Mg_2.3_Ge and Ca_2.4_Ge were calculated as −0.56 and −1.94 eV, respectively, indicating that Ca–Ge alloying was thermodynamically more stable than that of Mg–Ge. Notably, the formation energy for amorphous Mg_2.3_Ge had a positive value (+0.003 eV) when referenced to crystalline Ge. This result implies that the crystalline Ge electrode was less likely than the amorphous Ge electrode to form the Mg_2.3_Ge phase.

Table 1 shows the volumes of amorphous Mg*_x_*Ge, Ca*_x_*Ge, Li*_x_*Ge, Na*_x_*Ge, and K*_x_*Ge with full compositions. From the equation ([V(M*_x_*Ge) − V(Ge)]/*x*), the volumes occupied by M in M*_x_*Ge were 21.76, 34.88, 13.73, 29.94, and 63.32 Å^3^ for Mg*_x_*Ge, Ca*_x_*Ge, Li*_x_*Ge, Na*_x_*Ge, and K*_x_*Ge, respectively. The corresponding ratios for volume expansion (defined as (V − V_0_)/V_0_ × 100%) for Mg_2.3_Ge and Ca_2.4_Ge are 231% and 386% for Mg_2.3_Ge and Ca_2.4_Ge, respectively, as shown in Table 2. This result indicates that the volume expansion ratio was considerably smaller for the fully magnesiated Ge alloy than for the fully calciated Ge alloy. Another noteworthy point is that Mg_2.3_Ge showed a favorably lower volume expansion ratio (231%) compared with Li_4.0_Ge (253%). However, the volume expansion of magnesiated Ge could result in capacity degradation during cycling because the discharge–charge volume expansion ratio remains high. This challenge may be overcome by advanced electrode architectures, such as nanostructures, nanopores, and composite electrodes, as demonstrated in Ge anode systems [26,61,62,63,64]. Therefore, the calculated high capacity of 1697 mA·h·g^–1^ and low volume expansion ratio of 231% in the Mg_2.3_Ge alloy suggest that Mg_2.3_Ge could be utilized as a next-generation anode material in LIBs.

**Figure 3:**
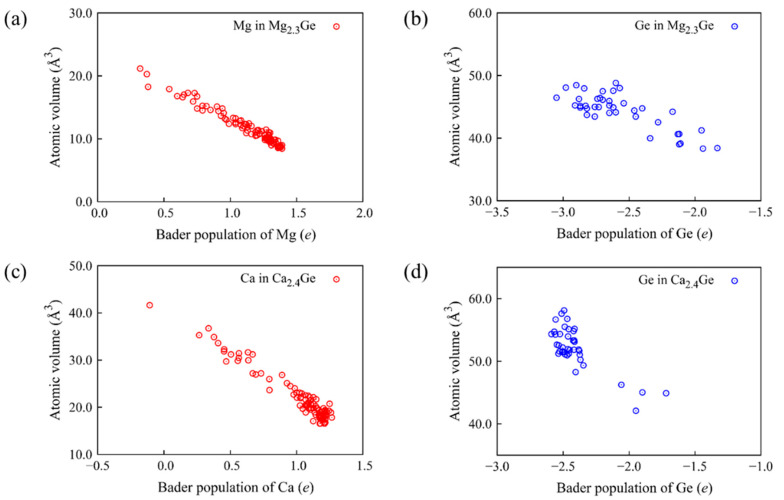
Relationship between atomic volume and charges for the elements (**a**) Mg and (**b**) Ge in Mg_2.3_Ge and (**c**) Ca and (**d**) Ge in Ca_2.4_Ge.

Figure 3 shows that the decrease in the charges of Mg, Ca, and Ge is closely related to the increase in their atomic volumes in amorphous Mg_2.3_Ge and Ca_2.4_Ge. These results indicate that the volume of M*_x_*Ge was determined by the total combined volume occupied by the M cations and Ge anions. The data points for Ge near −2.0 *e* are from isolated Ge–Ge pairs. While most of the Ge atoms are surrounded by the relatively larger Ca atoms with positive charges, the Ge atoms in the Ge–Ge pairs accept fewer electrons from Ca atoms, leading to the deviated data points shown in Figure 3d. The isolated host element clusters, such as the Ge–Ge pairs are usually observed in the amorphous structures with large alloying atoms [46]. We interpret the volume expansion of the amorphous M*_x_*Ge alloys as originating from (1) the ionic concentration, (2) the difference in atomic volume between M and Ge ions, and (3) the charge distribution between M and Ge ions.

**Figure 4:**
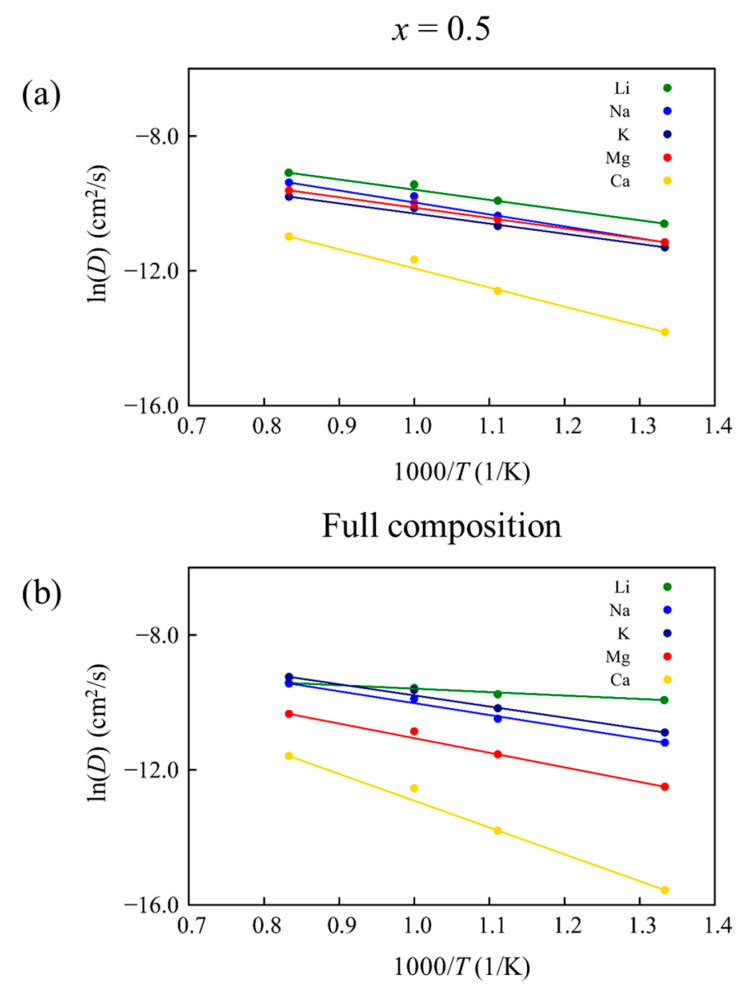
Logarithmic diffusivities (**a**) of carrier ions in M_0.5_Ge (M = Mg, Ca, Li, Na, and K) and (**b**) amorphous M*_x_*Ge (*x* = full composition).

We investigated the M-ion diffusivities in M_0.5_Ge amorphous alloys (M = Mg, Ca, Li, Na, and K) at T = 300 K, as shown in Table 2. Diffusivity studies using FPMD simulations can provide valuable information on the rate capability of Ge anodes in MIBs. The evaluated mean squared displacement (*d*_ms_) values linearly increased with time *t* for both Mg and Ca ions (Figure S2), thereby precisely determining the *D* values at those temperatures. The Arrhenius plots for all cations show linear variations in ln(*D*) with respect to the inverse temperature, as shown in Figure 4. The temperatures, at which the mean square displacements were computed were chosen carefully because calculations that were too high (i.e., above the melting point of 1211 K for Ge) could be problematic in terms of accuracy, and simulation times that were too close to room temperature could be extremely long. A further discussion is provided in the Supplementary Data section (Figure S2).

We suggest that Mg ions diffuse considerably more rapidly than Ca ions in M_0.5_Ge alloys at room temperature. The calculated ion diffusivities (*D*) are 3.0 × 10^–8^ and 1.1 × 10^–11^ cm^2^·s^–1^ for Mg ions in Mg_0.5_Ge and Ca ions in Ca_0.5_Ge at *T* = 300 K, respectively, as shown in Table 1. These results indicate that the Mg ion showed a diffusivity that was three orders of magnitude superior to that of the Ca ion. Notably, Mg ion diffusion occurred at a comparable order of magnitude in Mg_0.5_Ge and in the monovalent Li_0.5_Ge, Na_0.5_Ge, and K_0.5_Ge alloys. This result contradicts the prevailing view that the diffusion of multivalent ions is significantly lower than that of monovalent ions, as in the Ni_2_O_4_, Mn_2_O_4_, V_2_O_5_, and Ti_2_S_4_ systems. Furthermore, the Mg ion diffusivity in amorphous Ge was even one order of magnitude superior to the Mg ion diffusivity (2.3 × 10^–9^ cm^2^·s^–1^) in Mg_0.5_Si [47]. This difference from the common understanding may be due to the structural disorder of amorphous Ge. The ion diffusivity values for amorphous Mg_0.5_Ge, Ca_0.5_Ge, Li_0.5_Ge, Na_0.5_Ge, and K_0.5_Ge are shown in Table 1, where the ion diffusivity in amorphous systems has an order of Li > Mg > K > Na >> Ca.

Table 2 summarizes the performances of amorphous Mg*_x_*Ge, Ca*_x_*Ge Li*_x_*Ge, Na*_x_*Ge, and K*_x_*Ge. The specific capacity of Mg_2.3_Ge was somewhat lower (~4%) than that of Ca_2.4_Ge, which exhibited the highest capacity. However, Mg_2.3_Ge showed a relatively small volume expansion ratio (~150%) compared to Ca_2.4_Ge. In addition, the Mg ions in Mg_2.3_Ge diffused more rapidly than the Ca ions in Ca_2.4_Ge by three orders of magnitude. These findings suggest that the Mg–Ge alloys have significantly better cycling performance and rate capability than Ca–Ge alloys. Table 2 summarizes the performance of M*_x_*Ge (M = Mg, Ca, Li, Na, and K), showing that (1) the order of specific capacity is Ca > Mg > Li > Na > K, (2) the volume expansion ratio has the increasing order of Na < Mg < Li < K < Ca, and (3) the order of ion transport is Li > Mg > K > Na > Ca. Compared to alloys between Ge and monovalent ions, Ge–Mg alloys showed a moderately high specific capacity, low volume expansion ratio, and fast ion transport. Accordingly, Mg^2+^ is a more promising carrier ion than Ca^2+^ for the amorphous Ge electrode in MIBs because of its superior electrochemical properties that can even compete with monovalent ions.

We attempted to relate the structural properties in Mg_2.3_Ge and Ca_2.4_Ge to their specific capacities, volume expansion ratio, and ion diffusivities. We determined the specific capacities as 1697 for Mg*_x_*Ge and 1771 mA·h·g^–1^ for Ca*_x_*Ge from the highest concentration (*x*_max_ = 2.3 for Mg and 2.4 for Ca) at the most favorable formation energies as plotted in Figure 1. The detailed specific capacity (*C*) calculation is included in the Supporting Information. Note that *x*_max_ plays an important role in determining the specific capacities because both Mg*_x_*Ge and Ca*_x_*Ge have the same charge and molecular weight per structural formula unit.

**Figure 5:**
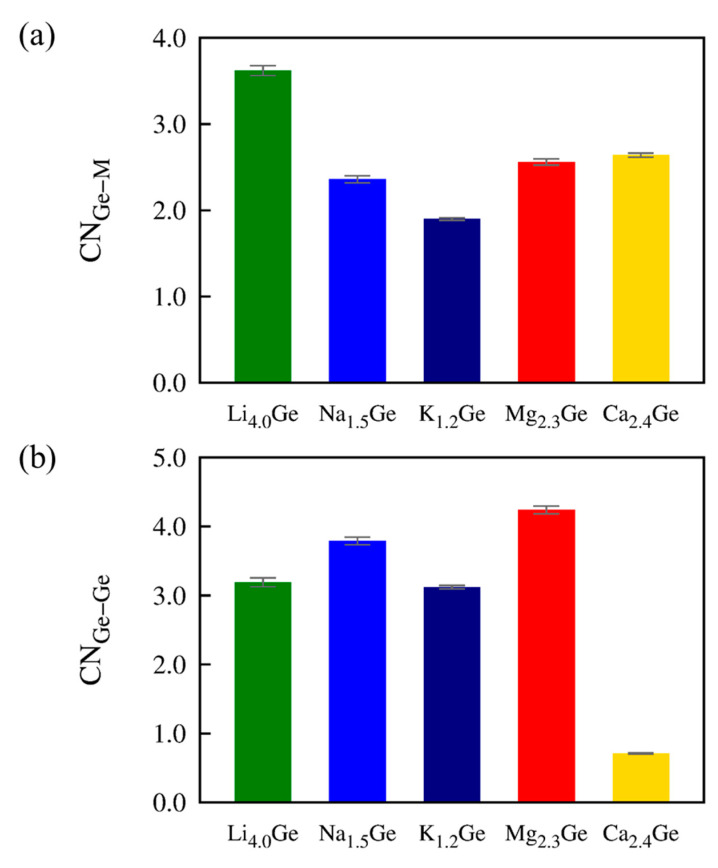
Average partial CN values of Ge in M*_x_*Ge (M= Mg, Ca, Li, Na, and K) (*x* = maximum concentration). (**a**) CN_Ge–M_ and (**b**) CN_Ge–Ge_. Cutoff distances for the nearest bond are 2.64, 3.11, 3.48, 2.79, 3.68, and 4.50 Å for Ge–M (M = Mg, Ca, Li, Na, and K) and Ge–Ge pairs, respectively (Figure S3 in the Supplementary data).

We found that the highest concentrations, *x*_max_, were related to the number of carrier ions that could be coordinated to the Ge host anions. The average host ion coordination numbers for Mg_2.3_Ge and Ca_2.4_Ge were CN_Ge–Mg_ = 2.56 and CN_Ge–Ca_ = 2.64, respectively, in line with a somewhat smaller capacity of Mg_2.3_Ge than of Ca_2.4_Ge. This relationship was consistent for monovalent ions as well as for these multivalent ions. Figure 5a shows that the coordination numbers are CN_Ge–Li_ = 3.62 in Li_4.0_Ge, CN_Ge–Na_ = 2.36 in Na_1.5_Ge, and CN_Ge–K_ = 1.90 in K_1.2_Ge. The CN_Ge–M_ (M = Mg, Ca, Li, Na, and K) values have the order Li (3.62) > Ca (2.64) > Mg (2.56) > Na (2.36) > K (1.90), which was analogous to the order of *x*_max_: Li (4.0) > Ca (2.4) > Mg (2.3) > Na (1.5) > K (1.2). Considering the different *n*_e_ for monovalent (1 × *x*_max_) and divalent (2 × *x*_max_) ions, we can obtain the order of capacity (Ca > Mg > Li > Na > K) from the order of maximum ion concentrations (*x*_max_). Furthermore, the CN values are CN_Ge–Ge_ = 4.24 in Mg_2.3_Ge and CN_Ge–Ge_ = 0.71 in Ca_2.4_Ge, as shown in Figure 5b. This result indicates that the Mg–Ge bond was weaker than the Ca–Ge bond and should result in rapid Mg ion transport in the Mg–Ge alloy.

The relationship between the volume extension ratios and the volumes accommodated by the M ions in M*_x_*Ge was also analyzed in terms of the *x*_max_ values. While the ion concentrations at the maximum charging for Mg*_x_*Ge (*x*_max_ = 2.3) and Ca*_x_*Ge (*x*_max_ = 2.4) were similar, a noticeable difference was observed in the volumes accommodated by Mg and Ca at 21.76 for Mg and 34.88 Å^3^ for Ca, resulting in a considerably lower volume expansion ratio of 231% for Mg_2.3_Ge compared to 386% for Ca_2.4_Ge. The contrasting difference in the volumes accommodated by Mg and Ca may originate from the difference in the atomic radii between Mg (1.50 Å) and Ca (1.80 Å). Notably, we found that the order of the volume occupied by M in M*_x_*Ge (M = Mg, Ca, Li, Na, and K) was K (63.32 Å^3^) > Ca (34.88 Å^3^) > Na (29.94 Å^3^) > Mg (21.76 Å^3^) > Li (13.73 Å^3^), which was consistent with the order of the atomic radii: K (2.20) > Ca (1.80) ≈ Na (1.80) > Mg (1.50) > Li (1.45 Å).

**Figure 6:**
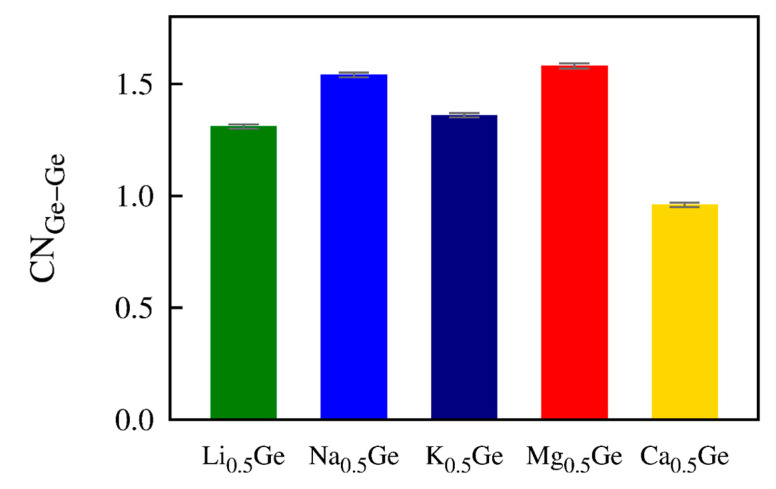
Average partial coordination numbers of Ge–Ge pairs in M_0.5_Ge (M= Mg, Ca, Li, Na, and K). The cutoff distances for the nearest bond are 2.64, 3.11, 3.48, 2.79, and 3.68 Å for Ge–Ge (M = Mg, Ca, Li, Na, and K) pairs, respectively.

Ion transport in amorphous alloys is a complicated process that is affected by the interaction between the carrier ions, the host ions, and the local structures [46,47]. In particular, the ion–host interaction, indicative of the M–Ge attraction (M = Mg, Ca, Li, Na, and K), appeared to be the primary reason for the considerably quicker ion diffusion in Mg_0.5_Ge (3.03 × 10^−8^ cm^2^·s^–1^) than in Ca_0.5_Ge (1.13 × 10^−11^ cm^2^·s^–1^). A weaker interaction between the carrier ions and the host ions creates conditions where ions can more easily break ion–host bonds and travel through the host structure. The strength of the M–Ge bond seemed to be related to the coordination number of Ge (CN_Ge–Ge_) in M*_x_*Ge at *x* = 0.5. The CN_Ge–Ge_ values (Figure 6) might indicate the weakness of the M–Ge bond, as a weak ion–Ge bond may prevent ions from breaking the Ge–Ge bond, allowing extra Ge–Ge bonds in the system. The coordination numbers were CN_Ge–Ge_ = 1.58 in Mg_0.5_Ge and CN_Ge–Ge_ = 0.96 in Ca_0.5_Ge, implying that the Mg–Ge bond was weaker than the Ca–Ge bond. This weak Mg–Ge bond can facilitate the diffusion of Mg ions.

The relationship between the M–Ge bond strength (M = Mg, Ca, Li, Na, and K) and the coordination number of CN_Ge–Ge_ could be applied to monovalent ion cases. The coordination numbers of CN_Ge–Ge_ were 1.58, 0.96, 1.31, 1.54, and 1.36 in Mg*_x_*Ge, Ca*_x_*Ge, Li*_x_*Ge, Na*_x_*Ge, and K*_x_*Ge (*x* = 0.5), respectively, implying that the bond strength of M–Ge (M = Mg, Ca, Li, Na, and K) follows the order of Ca > Li > K > Na ~ Mg, consistent with the order of the thermodynamic driving forces for alloy formation (Ca > Li > K > Mg > Na). Assuming that the interaction between ion and host atoms is the primary factor that determines the formation energy of M*_x_*Ge, the observed analogous trends strongly support the discussion that the strength of the bond between ion and host Ge atoms is highly correlated with the CN_Ge–Ge_ values. Along with the aforementioned discussion on CN_Ge–M_, we conclude that the CN_host–host_ and CN_host–ion_ values can be used as important indicators for evaluating the electrochemical performance of alloy-based anode materials, including the formation energy, specific capacity, volume expansion ratio, and ion diffusivity.

## 4. Conclusions

By investigating multivalent ions such as Al^3+^, Mg^2+^, Ca^2+^, Zn^2+^, and using first-principles calculations, we demonstrated that Mg^2+^ and Ca^2+^ can work stably as multivalent carrier ions for amorphous Ge anodes. Furthermore, Mg^2+^ has better cycle stability and rate capability than Ca^2+^. Specifically, Mg_2.3_Ge demonstrates a capacity of 1697 mA·h·g^–1^, a volume expansion ratio of 231%, and an ion diffusivity of 3.0 × 10^–8^ cm^2^·s^–1^; these values are equivalent to or superior to those of Li_4.0_Ge, Na_1.5_Ge, and K_1.2_Ge. The strong correlation between the electrochemical performance and structural properties of Ge alloys with monovalent and multivalent ions offers comprehensive insight into alloying mechanisms. The findings of this study suggest that Ge anodes could be used in practical multivalent-ion batteries, as Mg^2+^ emerged as the most promising multivalent ion for amorphous Ge anodes and showed comparable or even better performance than traditional monovalent ions. Moreover, we also found that the CN values of the host atoms (CN_host–host_) and ions (CN_host–ion_) can be used to understand the relationship between the structural properties and electrochemical performance of alloying anode materials. The results presented here will serve as a helpful reference for experimental researchers working on the development of alloying anode materials for multivalent-ion batteries. Moreover, our results demonstrate a new class of materials that can overcome the skyrocketing demands for battery capacities while also revealing an effective theoretical methodology for the development of alloying electrodes, which are among the most promising electrode materials for next-generation batteries [65,66].

## Figures and Tables

**Table 1:** Diffusion parameters of M_0.5_Ge (M = Li, Na, K, Mg, and Ca) *^a^*.

Carrier Ion	*E*_D_	*D*_0_	*D*
Li	0.26	1.4 × 10^−3^	5.9 × 10^−8^
Na	0.31	1.7 × 10^−3^	1.2 × 10^−8^
K	0.26	7.0 × 10^−4^	2.8 × 10^−9^
Mg	0.27	8.7 × 10^−4^	3.0 × 10^−8^
Ca	0.49	1.9 × 10^−3^	1.1 × 10^−11^

*^a^ E*_D_ (eV), *D*_0_ (cm^2^ s^–1^), and *D* (cm^2^ s^–1^) are the activation energy for diffusion, the pre-exponential factor, and the self-diffusion coefficient at *T* = 300 K, respectively.

**Table 2:** Capacities (mA h g^–1^) and expansion ratios in volume (%), diffusivities (cm^2^ s^–1^) at *x* = 0.5 in M*_x_*Ge (M = Mg, Ca, Li, Na, and K) of Li_4.0_Ge, Na_1.5_Ge, K_1.2_Ge, Mg_2.3_Ge, and Ca_2.4_Ge.

Carrier Ion	Capacity	Expansion Ratio	Diffusivity
Li	1476	253	5.9 × 10^−8^
Na	553	207	1.2 × 10^−8^
K	443	351	2.8 × 10^−8^
Mg	1697	231	3.0 × 10^−8^
Ca	1771	389	1.1 × 10^−11^

## Data Availability

The data that support the findings of this study are available from the corresponding authors upon reasonable request.

## Supplementary Materials

The following supporting information can be downloaded at: https://www.mdpi.com/article/10.3390/nano13212868/s1, Figure S1: Temperature and energy changes during the FPMD simulation of Mg_2.3_Ge; Figure S2: Mean squared displacements for Mg and Ca atoms; Figure S3: Partial radial distribution functions g(r) for Ge–Ge pairs; Figure S4: Average partial coordination numbers, CN_Ge–M_; Figure S5: The average voltage of M*_x_*Ge; Table S1: Atomic coordinates of Mg and Ge atoms in Mg_2.3_Ge; Table S2: Atomic coordinates of Ca and Ge atoms in Ca_2.4_Ge. Refs. [37,45,46,49,67] are cited in Supplementary Materials.
