# Supplementary material for: First-Principles Dynamics Investigation of Germanium as an Anode Material in Multivalent-Ion Batteries
Source: Nanomaterials (Basel). 2023 Oct 30;13(21):2868. doi: 10.3390/nano13212868 (PMC10650491; doi:10.3390/nano13212868)
Supplement: Supplementary file 1 [file nanomaterials-13-02868-s001.zip › nanomaterials-2672036-supplementary.pdf]

Supplementary data for

First-Principles Dynamics Investigation of Germanium as an Anode Material in Multivalent-Ion Batteries

Chaewon Kim, Useul Hwang, Sangjin Lee * and Young-Kyu Han *

Department of Energy and Materials Engineering, Dongguk University-Seoul, Seoul 04620, Republic of Korea

* Correspondence: dltkdwls@dongguk.edu (S.L.); ykenergy@dongguk.edu (Y.-K.H.)

FPMD simulations for generating amorphous structures

The average volume of five optimized M_xGe structures was computed to determine the final volume. After determining the final volume of M_xGe, we calculated the energy of M_xGe using this volume. To do this, we adjusted the five final optimized structures in the volume relaxation step to a cubic supercell with the specifically determined volume. We then equilibrated the structure for 5–10 ps at 300 K (Figure S1) until the energy in the FPMD simulation remained consistent (Figure S1). We used static DFT calculations to calculate the energies of five structures that were selected every 0.5 ps during the last 2 ps of the FPMD run (Figure S1). We averaged the energies of five structures to evaluate the final energy of A_xGe, which was used to compute the formation energy of M_xGe. These FPMD simulations using the liquid-quench method for constructing amorphous structures has been successfully demonstrated in our previous studies on amorphous Li_xSi [1], Na_xSi [2], K_xSi [3], $Na_xAl_2O_3$ [4], and Sn_4P_3 [5].

We performed the FPMD simulations for 10 ps at higher temperatures (T = 900, 1050, 1200, and 1350 K) to effectively evaluate the diffusivity at T = 300 K, then the ion diffusivities at each temperatures (≥ 900 K) were analyzed based on the Einstein relation $D = \langle r^2(t) \rangle / 6t$, where D and $\langle r^2 \rangle$ represent the diffusivity and mean square displacement (d_{ms}), respectively. The D value at T = 300 K was extrapolated using the determined D values at high temperatures based on the Arrhenius relation $D = D_0 \exp(-E_D/k_B T)$, where D_0, E_D, and k_B are the pre-exponential factor, activation energy for diffusion, and Boltzmann constant, respectively.

The specific capacity (C) of the electrodes was evaluated using the equation, $C = n_e F / M_w$, where n_e, F, and M_w represent the number of electrons transferred per structural formula unit, the Faraday constant, and the molecular weight per structural formula unit.

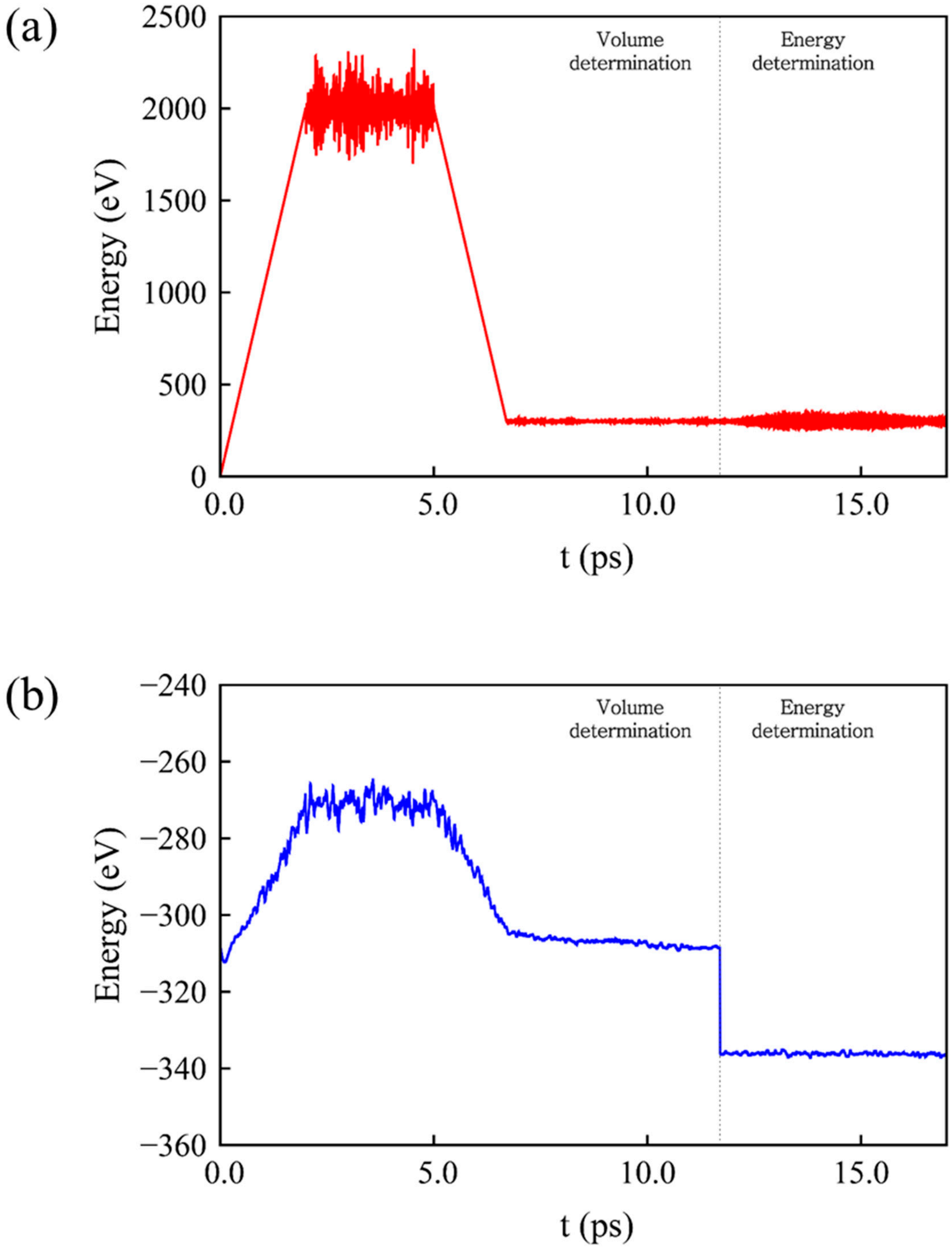

Figure S1. (**a**) Temperature and (**b**) energy changes in the volume and energy determination stages during the FPMD simulation of $Mg_{2.3}Ge$. In (**b**), the arrows represent the five selected structures to determine the volume or energy of $Mg_{2.3}Ge$.

Diffusion computation for amorphous $Mg_{2.3}Ge$ and $Ca_{2.4}Ge$

We computed the mean square distribution (MSD) of Mg, Ca, and Ge in amorphous Mg_xGe and Ca_xGe using the first-principles molecular dynamics (FPMD) simulations. Using the optimal atomic coordinates obtained from the simulations 25–30 ps at T = 750, 900, 1050, and 1200 K (Figure S2), the self-diffusion coefficient D values were estimated using the Einstein relation $\langle r^2(t) \rangle = 6Dt$, $\langle r^2(t) \rangle$ is the MSD. The extrapolated D values at T = 300 K were computed using the D values at high temperatures used in Figure S1, based on the Arrhenius law $D = D_0 \exp(-E_D/k_BT)$, where D_0 is the pre-exponential factor, E_D is the activation energy for self-diffusion, and k_B is the Boltzmann constant.

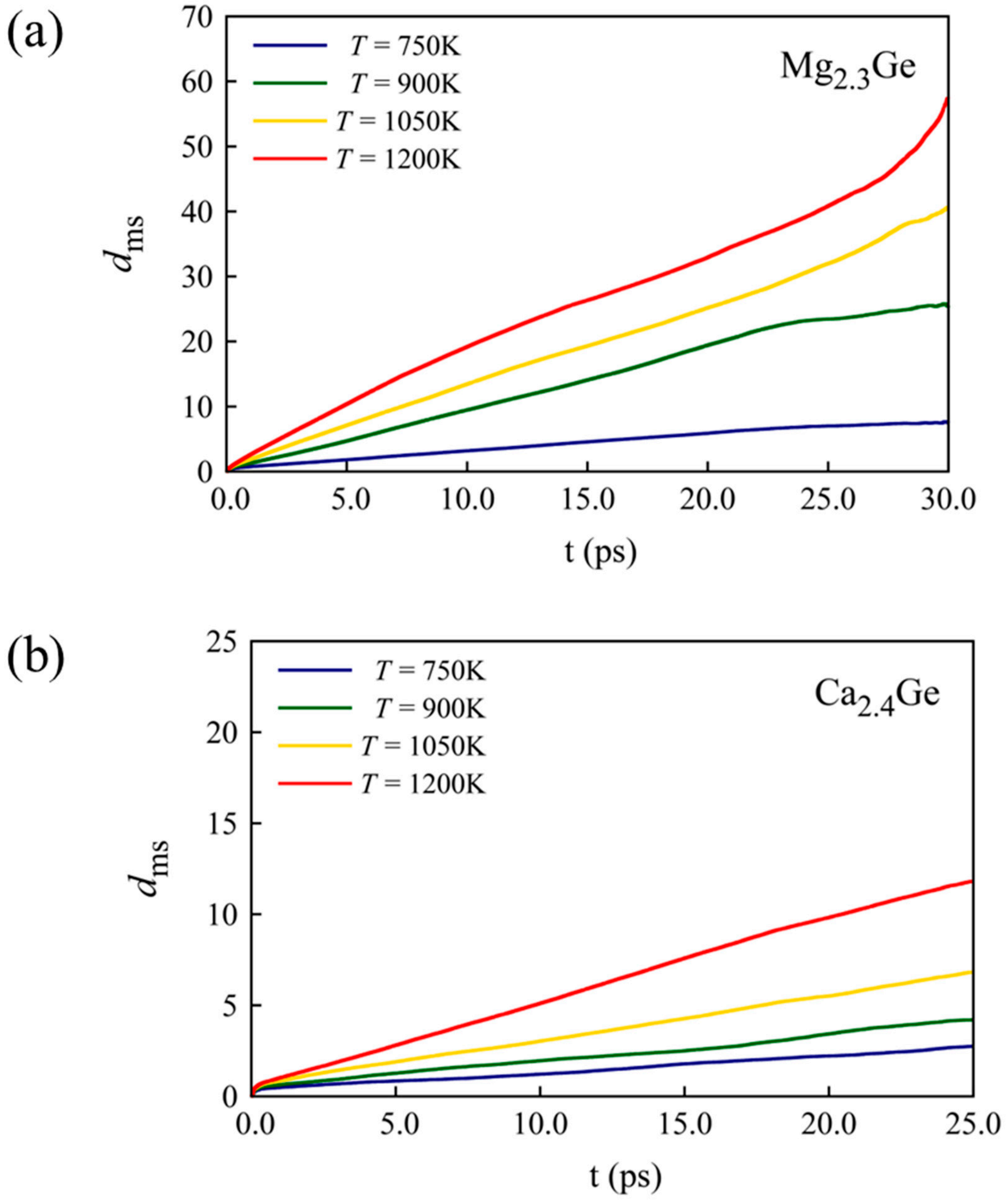

Figure S2. Mean squared displacements at 750, 900, 1050, and 1200 K for (**a**) Mg and (**b**) Ca atoms in amorphous $Mg_{2.3}Ge$ and $Ca_{2.4}Ge$, respectively.

Structural analyses of amorphous M_xGe (M = Li, Na, K, Mg, and Ca)

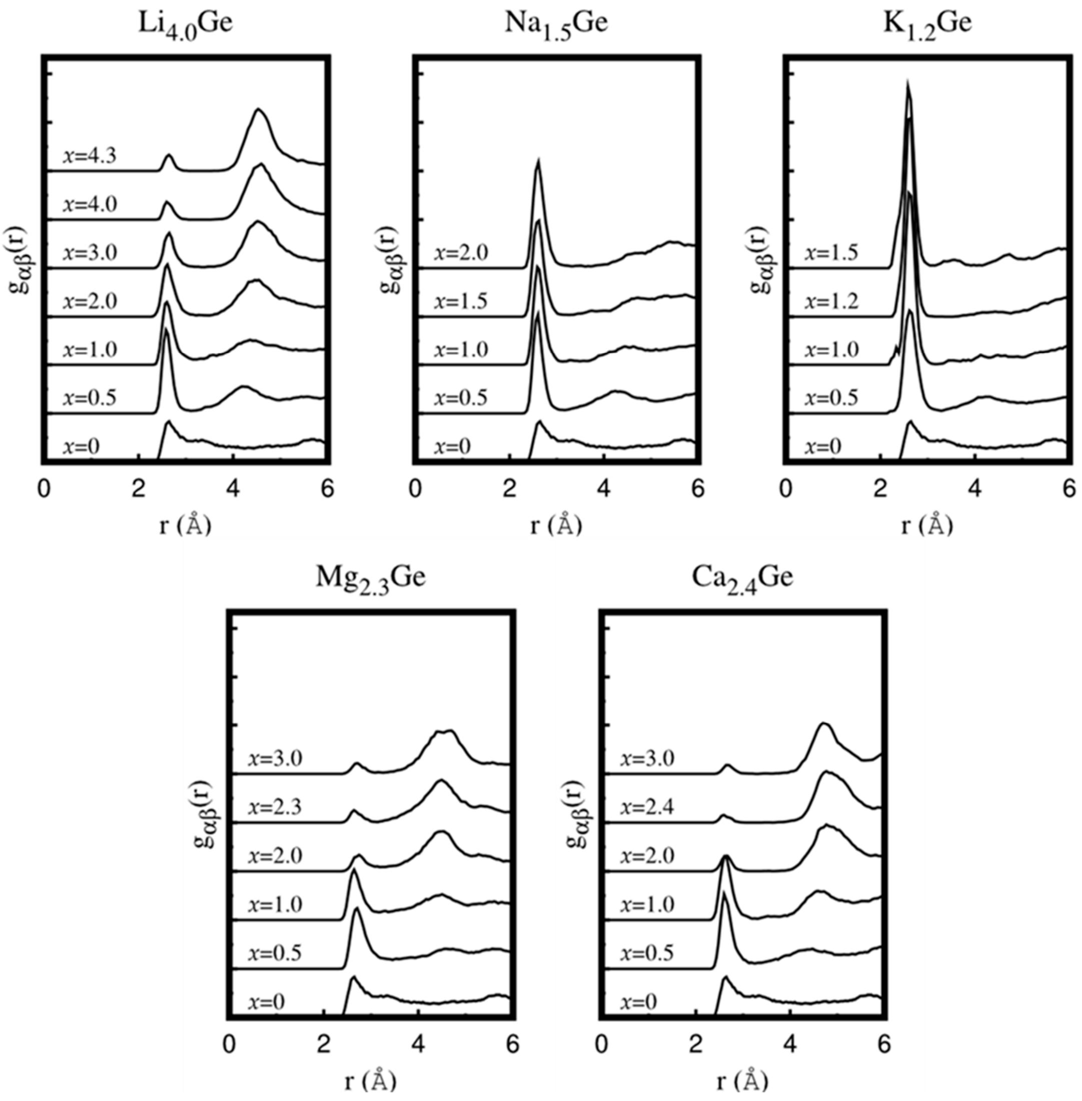

Figure S3. Partial radial distribution functions g(r) for Ge–Ge pairs in amorphous Li_xGe, Na_xGe, K_xGe, Mg_xGe, Ca_xGe.

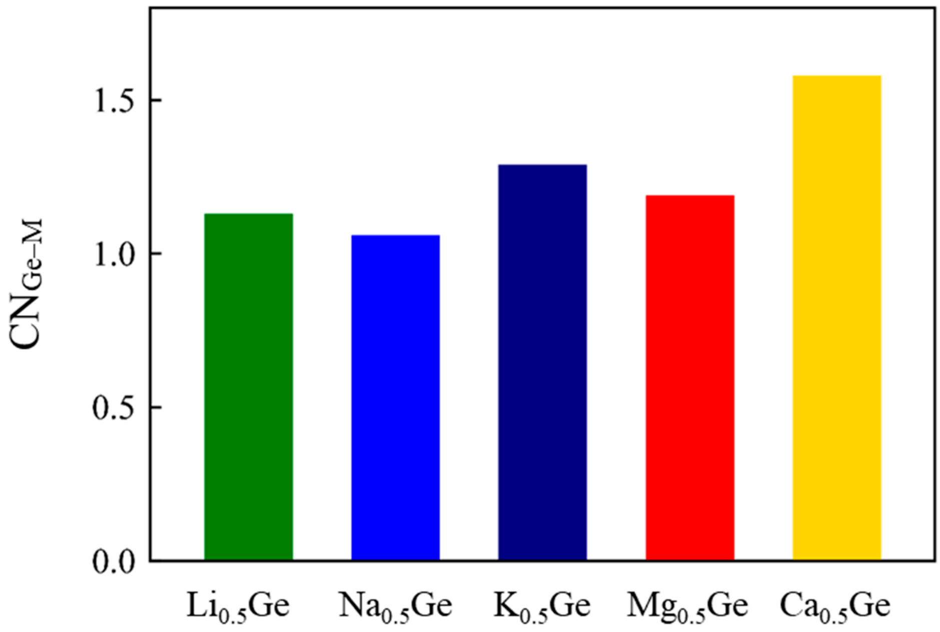

Figure S4. Average partial coordination numbers, $CN_{Ge–M}$ (A = Li, Na, K, Mg, and Ca), of Ge in amorphous $Li_{0.5}Ge$, $Na_{0.5}Ge$, $K_{0.5}Ge$, $Mg_{0.5}Ge$, and $Ca_{0.5}Ge$.

Average voltage for magnesiation and calciation of amorphous Ge anodes

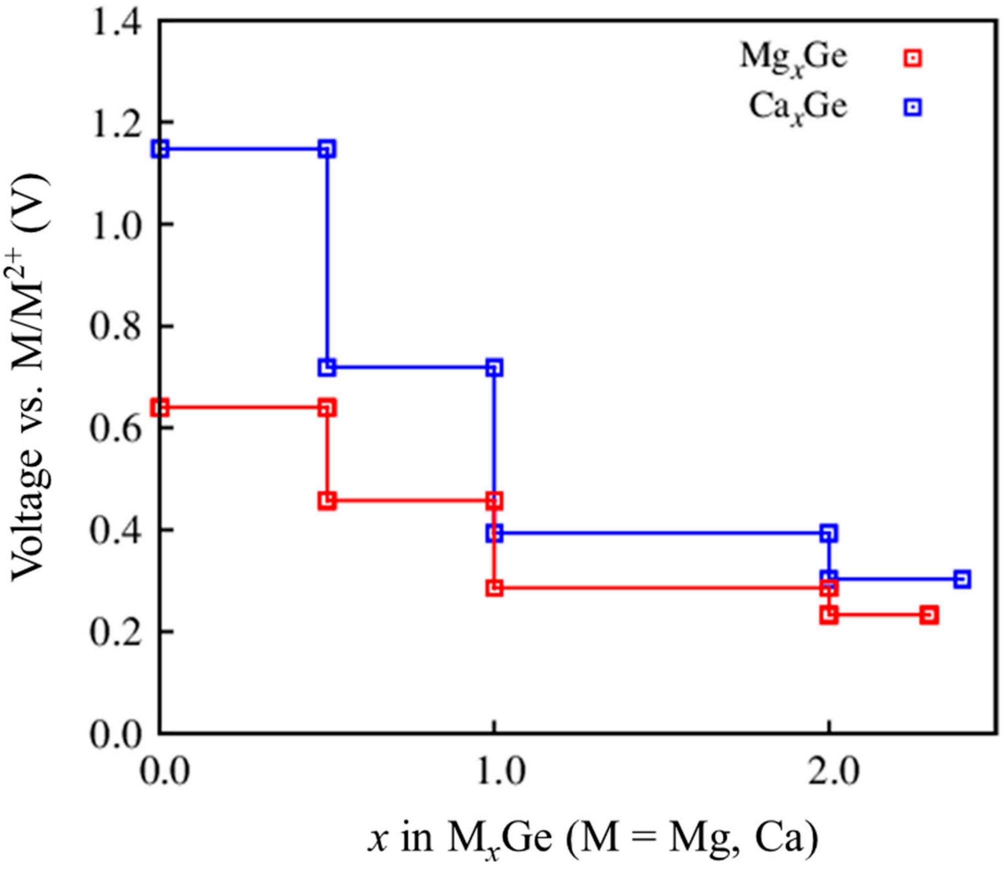

Figure S5. The average voltages of M_xGe (M = Mg and Ca) defined as V(x) = –[E_{tot}($M_{x+\Delta x}$Ge) – E_{tot}(M_xGe)]/(2eΔx) + E_{tot}(M).

Table S1. Atomic coordinates of Mg and Ge atoms in amorphous $Mg_{2.3}Ge$ with a cubic unit cell; lattice parameter is 14.20946 Å.

Atom	x	y	z	Atom	x	y	z
Mg	0.945015	0.861094	0.179767	Mg	0.408474	0.472042	0.655872
Mg	0.987043	0.068989	0.114098	Mg	0.216891	0.028543	0.535191
Mg	0.011274	0.502462	0.173294	Mg	0.543605	0.070096	0.287605
Mg	0.776861	0.020728	0.194514	Mg	0.337087	0.827239	0.526662
Mg	0.836057	0.561939	0.998952	Mg	0.707038	0.259957	0.573919
Mg	0.255033	0.562398	0.236949	Mg	0.556869	0.570387	0.465225
Mg	0.607855	0.555636	0.002890	Mg	0.178491	0.940513	0.182522
Mg	0.919452	0.241805	0.678172	Mg	0.833906	0.926314	0.743920
Mg	0.327340	0.241852	0.035672	Mg	0.927485	0.394388	0.862754
Mg	0.096021	0.879137	0.784126	Mg	0.862624	0.981139	0.963591
Mg	0.636982	0.707800	0.295907	Mg	0.978928	0.012461	0.583591
Mg	0.669043	0.939087	0.387132	Mg	0.469756	0.859815	0.704896
Mg	0.980595	0.778668	0.393709	Mg	0.729864	0.473332	0.603037
Mg	0.544461	0.256014	0.390288	Mg	0.426175	0.063902	0.915306
Mg	0.455676	0.825623	0.314037	Mg	0.932213	0.752510	0.892946
Mg	0.055758	0.856482	0.997836	Mg	0.942662	0.518053	0.405020
Mg	0.757188	0.143631	0.784406	Mg	0.789679	0.450745	0.176739
Mg	0.113270	0.404197	0.326038	Mg	0.902384	0.178481	0.471194
Mg	0.340313	0.167899	0.235049	Mg	0.372689	0.075980	0.701419
Mg	0.358960	0.722140	0.141959	Mg	0.069417	0.365056	0.530850
Mg	0.580600	0.036083	0.588717	Mg	0.336543	0.627592	0.457263

Atom	x	y	z	Atom	x	y	z
Mg	0.563828	0.791094	0.502152	Mg	0.815304	0.853269	0.538532
Mg	0.130400	0.606473	0.525964	Mg	0.220663	0.037425	0.989635
Mg	0.128475	0.115722	0.720373	Mg	0.427025	0.686515	0.935776
Mg	0.888293	0.334726	0.322165	Mg	0.332168	0.539917	0.844498
Mg	0.120059	0.240402	0.937657	Mg	0.107634	0.120102	0.346593
Mg	0.631363	0.733828	0.847633	Mg	0.956099	0.472487	0.666158
Mg	0.271800	0.711002	0.710323	Mg	0.592991	0.507010	0.226693
Mg	0.645959	0.974747	0.025535	Mg	0.409931	0.433332	0.388491
Mg	0.622456	0.652700	0.658587	Mg	0.916514	0.980188	0.374757
Mg	0.318496	0.880916	0.869922	Mg	0.185176	0.710052	0.912171
Mg	0.528488	0.307386	0.143599	Mg	0.205487	0.766134	0.351892
Mg	0.140803	0.713595	0.134476	Mg	0.195490	0.412125	0.088313
Mg	0.041463	0.265652	0.173531	Mg	0.805003	0.248138	0.143822
Mg	0.049750	0.527739	0.979479	Mg	0.395089	0.491748	0.113093
Mg	0.254944	0.271033	0.421356	Mg	0.306948	0.980773	0.336614
Mg	0.068933	0.658694	0.745483	Mg	0.715867	0.460257	0.419159
Mg	0.422891	0.941967	0.139108	Mg	0.454221	0.239596	0.595932
Mg	0.552189	0.749513	0.092995	Mg	0.355369	0.325213	0.834721
Mg	0.773017	0.802786	0.113154	Mg	0.584949	0.198655	0.951853
Mg	0.764580	0.654680	0.460202	Mg	0.724776	0.527281	0.822110
Mg	0.718212	0.363776	0.957489	Mg	0.129540	0.824618	0.578708

Atom	x	y	z	Atom	x	y	z
Mg	0.179543	0.480430	0.690928	Ge	0.774252	0.348231	0.754970
Mg	0.821962	0.693730	0.697504	Ge	0.917894	0.580309	0.822699
Mg	0.903112	0.196829	0.940191	Ge	0.264751	0.592225	0.041076
Mg	0.631917	0.967465	0.809882	Ge	0.678324	0.837488	0.667868
Ge	0.505517	0.386131	0.959078	Ge	0.101574	0.178740	0.528403
Ge	0.384398	0.131880	0.445126	Ge	0.114254	0.934113	0.390850
Ge	0.867434	0.377114	0.513432	Ge	0.273561	0.161027	0.855854
Ge	0.759409	0.846342	0.915235	Ge	0.172723	0.133818	0.149243
Ge	0.671911	0.341352	0.270482	Ge	0.528830	0.542970	0.832055
Ge	0.038058	0.051560	0.916477	Ge	0.551716	0.384771	0.533329
Ge	0.767757	0.062917	0.519919	Ge	0.810386	0.816382	0.324762
Ge	0.450784	0.686493	0.606680	Ge	0.085710	0.608439	0.320808
Ge	0.938431	0.077535	0.761272	Ge	0.454206	0.959367	0.469696
Ge	0.963120	0.813311	0.654575	Ge	0.186411	0.416211	0.890317
Ge	0.927111	0.372615	0.053490	Ge	0.614807	0.891827	0.214446
Ge	0.912663	0.150138	0.275251	Ge	0.084486	0.331041	0.753568
Ge	0.252232	0.859354	0.036334	Ge	0.441226	0.596872	0.291128
Ge	0.501163	0.863040	0.928755	Ge	0.485198	0.129597	0.104719
Ge	0.943083	0.644956	0.545615	Ge	0.713108	0.626557	0.141025
Ge	0.965293	0.695710	0.078761	Ge	0.552835	0.144486	0.770378
Ge	0.746941	0.137190	0.002286	Ge	0.251784	0.442966	0.513250

Table S2. Atomic coordinates of Ca and Ge atoms in amorphous $Ca_{2.4}Ge$ with a cubic unit cell; lattice parameter is 16.15366 Å.

Atom	x	y	z	Atom	x	y	z
Ca	0.651505	0.898526	0.83918	Ca	0.95407	0.330298	0.360609
Ca	0.328615	0.835629	0.489011	Ca	0.834373	0.845186	0.156809
Ca	0.497905	0.038764	0.210897	Ca	0.967171	0.311039	0.087834
Ca	0.751924	0.56257	0.91837	Ca	0.540986	0.606161	0.008833
Ca	0.750332	0.53711	0.693754	Ca	0.246551	0.096354	0.597775
Ca	0.174309	0.692687	0.433394	Ca	0.101544	0.89443	0.570993
Ca	0.775067	0.997437	0.994097	Ca	0.047898	0.73435	0.24694
Ca	0.61212	0.589932	0.561623	Ca	0.902633	0.923277	0.802349
Ca	0.534353	0.540858	0.789296	Ca	0.728028	0.217688	0.407962
Ca	0.723265	0.592985	0.384757	Ca	0.910657	0.074133	0.163098
Ca	0.445095	0.971453	0.597717	Ca	0.721605	0.354155	0.036206
Ca	0.001819	0.402299	0.678163	Ca	0.43492	0.403831	0.573345
Ca	0.442192	0.287001	0.369968	Ca	0.285357	0.608725	0.955941
Ca	0.480055	0.289637	0.127296	Ca	0.826795	0.787143	0.397032
Ca	0.224492	0.318372	0.064839	Ca	0.555415	0.127309	0.477484
Ca	0.200025	0.612928	0.149994	Ca	0.401842	0.193191	0.704319
Ca	0.314492	0.499218	0.366814	Ca	0.527501	0.091007	0.908438
Ca	0.909164	0.837808	0.592073	Ca	0.847217	0.465645	0.5142
Ca	0.617363	0.390245	0.273739	Ca	0.910147	0.657644	0.071563
Ca	0.12634	0.394831	0.24412	Ca	0.319361	0.288274	0.870144
Ca	0.066958	0.183544	0.260151	Ca	0.724283	0.559647	0.151954
Ca	0.376407	0.995992	0.033528	Ca	0.958056	0.625431	0.41423
Ca	0.796775	0.249332	0.233853	Ca	0.210411	0.121765	0.950252

Atom	x	y	z	Atom	x	y	z
Ca	0.374394	0.475608	0.091091	Ca	0.184305	0.696081	0.664221
Ca	0.333436	0.839266	0.697889	Ca	0.647723	0.390535	0.510393
Ca	0.953847	0.954628	0.355976	Ca	0.296849	0.502588	0.757034
Ca	0.106631	0.474992	0.48646	Ca	0.520789	0.360157	0.929188
Ca	0.584738	0.287878	0.71522	Ca	0.152435	0.016756	0.11953
Ca	0.185382	0.780602	0.868056	Ca	0.680399	0.808922	0.537785
Ca	0.656341	0.757372	0.249929	Ca	0.31794	0.010732	0.81528
Ca	0.34708	0.046606	0.387565	Ca	0.879041	0.397968	0.888952
Ca	0.495161	0.577264	0.223185	Ca	0.496673	0.828316	0.146438
Ca	0.61574	0.060257	0.707555	Ca	0.240962	0.800879	0.083853
Ca	0.300027	0.163037	0.179118	Ca	0.77631	0.733936	0.770834
Ca	0.229629	0.259194	0.405265	Ca	0.97354	0.632171	0.649889
Ca	0.438271	0.810953	0.91457	Ca	0.071363	0.110343	0.462475
Ca	0.100942	0.551088	0.826535	Ca	0.842452	0.099642	0.705829
Ca	0.784594	0.319025	0.699603	Ca	0.225943	0.33672	0.639222
Ca	0.717228	0.025271	0.291006	Ca	0.056539	0.17783	0.684172
Ca	0.028266	0.874895	0.99948	Ca	0.111861	0.973924	0.779632
Ca	0.489394	0.671328	0.408188	Ca	0.328609	0.773662	0.271348
Ca	0.966578	0.703166	0.871167	Ca	0.928728	0.498038	0.225327
Ca	0.099783	0.304055	0.86946	Ca	0.534521	0.895085	0.393253
Ca	0.533089	0.771144	0.72735	Ca	0.96677	0.141303	0.94096
Ca	0.057441	0.49797	0.012331	Ca	0.162458	0.916754	0.324913
Ca	0.745521	0.209944	0.874889	Ca	0.675333	0.774374	0.021328

Atom	x	y	z	Atom	x	y	z
Ca	0.774936	0.014319	0.523348	Ca	0.355167	0.691463	0.790697
Ca	0.643564	0.139749	0.092417	Ca	0.474856	0.941887	0.782352
Ca	0.389578	0.635785	0.621402	Ca	0.69276	0.398991	0.839846
Ca	0.900858	0.203212	0.527776	Ca	0.706087	0.198845	0.592645
Ca	0.82148	0.18698	0.056891	Ca	0.851911	0.822619	0.961872
Ca	0.495324	0.788285	0.556285	Ca	0.37836	0.224119	0.529273
Ca	0.143923	0.515269	0.653835	Ca	0.903215	0.549964	0.790953
Ca	0.081908	0.289601	0.526182	Ca	0.385787	0.184191	0.99881
Ca	0.781005	0.407062	0.357362	Ca	0.317263	0.351626	0.23246
Ca	0.372157	0.671468	0.103617	Ca	0.213905	0.438633	0.933911
Ca	0.328384	0.964308	0.21968	Ca	0.093538	0.686338	0.006568
Ca	0.212811	0.172924	0.775271	Ca	0.020448	0.921681	0.195367
Ca	0.432735	0.365394	0.763493	Ca	0.61716	0.687974	0.863576
Ca	0.739188	0.915247	0.684632	Ca	0.843779	0.668518	0.242638
Ca	0.657151	0.926729	0.154281	Ca	0.264988	0.509685	0.555936
Ca	0.090483	0.186765	0.07629	Ca	0.808809	0.658023	0.547981
Ca	0.870729	0.468996	0.058933	Ca	0.933708	0.252083	0.789739
Ca	0.217246	0.936937	0.940844	Ca	0.025424	0.800024	0.435385
Ca	0.045166	0.802211	0.743892	Ca	0.571736	0.447514	0.09087
Ca	0.106643	0.560754	0.322337	Ca	0.885217	0.135022	0.353788
Ca	0.570762	0.951127	0.021511	Ca	0.610595	0.204583	0.272313
Ca	0.97864	0.030249	0.606103				
Ca	0.503809	0.490654	0.415581				

References

[1] Jung, S. C.; Choi, J. W.; Han, Y.-K. *Nano Lett.* **2012**, *12*, 5342–5347.

[2] Jung, S. C.; Jung, D. S.; Choi, J. W.; Han, Y.-K. *J. Phys. Chem. Lett.* **2014**, *5*, 1283–1288.

[3] Lee, S.; Jung, S. C.; Han, Y.-K. *J. Power Sources* **2019**, *415*, 119–125.

[4] Jung, S. C.; Kim, H.-J.; Choi, J. W.; Han, Y.-K. *Nano Lett.* **2014**, *14*, 6559–6563.

[5] Jung, S. C.; Choi, J.-H.; Han, Y.-K. *J. Mater. Chem. A* **2018**, *6*, 1772–1779.
